# Cognitive Behavioral Approach to Treat Obesity: A Randomized Clinical Trial

**DOI:** 10.3389/fnut.2021.611217

**Published:** 2021-02-18

**Authors:** Amanda dos Santos Moraes, Ricardo da Costa Padovani, Cauê Vazquez La Scala Teixeira, Maria Gabriela Soria Cuesta, Silvandro dos Santos Gil, Bárbara de Paula, Gilberto Monteiro dos Santos, Rodrigo Tributino Gonçalves, Ana Raimunda Dâmaso, Lila Missae Oyama, Ricardo José Gomes, Danielle Arisa Caranti

**Affiliations:** ^1^Post Graduate Program of Interdisciplinary Health Sciences, Federal University of São Paulo, São Paulo, Brazil; ^2^Obesity Study Group (GEO), Post-Graduate Program of Nutrition, Federal University of São Paulo, São Paulo, Brazil; ^3^Health, Education and Society Department, Federal University of São Paulo, São Paulo, Brazil; ^4^Post Graduate Program of Food, Nutrition, Federal University of São Paulo, São Paulo, Brazil; ^5^Graduate Program of Interdisciplinary Health Sciences, Federal University of São Paulo, São Paulo, Brazil; ^6^Institute of Mathematical and Computer Sciences, University of São Paulo, São Carlos, Brazil; ^7^Biosciences Department, Federal University of São Paulo, São Paulo, Brazil

**Keywords:** cognitive behavioral therapy for obesity, obesity and comorbidities, psychological factors of obesity, psychological factors of weight loss, quality of life

## Abstract

Our aim was to analyze and compare the effects of three different long-term treatments on anthropometric profiles, eating behaviors, anxiety and depression levels, and quality of life of groups of adults with obesity.

**Methods:** The 43 participants in the study were randomly assigned to one of three groups: the education and health group (EH, *n* = 12), which received lectures on health topics; the physical exercise group (PE, *n* = 13), which underwent physical training; and the interdisciplinary therapy plus cognitive behavioral therapy (IT + CBT) (*n* = 18) group, which received physical training, nutritional advice, and physical and psychological therapy.

**Results:** Total quality of life increased significantly in the EH group (△ = 2.00); in the PE group, body weight significantly decreased (△ = −1.42) and the physical domain of quality of life improved (△ = 1.05). However, the most significant changes were seen in the IT + CBT group, in which the anthropometric profile improved; there were an increase in quality of life in all domains (physical, psychological, social, and environmental), an improvement in eating behaviors [Dutch Eating Behavior Questionnaire (DEBQ), total △ = −8.39], and a reduction in depression [Beck Depression Inventory (BDI), △ = −10.13).

**Conclusion:** The IT + CBT program was more effective than the PE and EH programs.

**Clinical Trial Registration Number:** NCT02573688.

## 1. Introduction

Over the past ~50 years, the prevalence of obesity has increased worldwide to pandemic proportions (1). It is increasingly linked with high levels of morbidity and mortality in associated diseases (2).

In Brazil, as in many countries, treatments for obese individuals that deal with the consequences of the condition represent an enormous expenditure for the health system. The costs of procedures associated with overweight and obesity in Brazil are an estimated 2.1 billion dollars per year (3).

A recent meta-analysis (4) that examined the worldwide prevalence of attempts to control weight (72 studies; *n*= 1,189,942) showed that 42% of the general population of adults were trying to lose weight, and 23% were trying to maintain their weight (5).

The most commonly accepted paradigm in relation to weight loss has been that an imbalance between food intake and physical activity is the main cause of overweight and obesity. However, this simple view does not take into account many other factors related to the problem, such as the influence of the modern lifestyle that stimulates over eating, or the role of adipose tissue in body homeostasis and energy balance. These complexities prevent a simple reductionist approach that often results in ineffective weight loss programs (6, 7).

There is a consensus in literature that the etiology of obesity is quite complex, presenting a multifactorial character. It therefore involves a range of factors, biological, psychosocial, and behavioral, which include genetic makeup, socioeconomic status, and cultural influences (8).

Kolotkin and Andersen (9) report that obesity has a substantial impact on a person's health-related quality of life (HRQOL). In this context, quality of life and cognitive variables are important elements to consider in respect of lasting positive results in the long-term treatment of obesity (10) (see Table 1). Cognitive behavioral therapy (CBT) has been demonstrated to be the most preferred intervention for obesity among overweight individuals (11), and it is one of the most commonly used psychological approaches. Patients benefit greatly from CBT as it can help to improve psychological skills, enabling stimulus control and a reduction in the quantities of food consumed. Learning behavioral modification strategies such as taking time to savor food, chewing slowly, and attaining a greater awareness of the pleasure of food that is associated with taste can also assist in weight loss (5).

**Table 1:** Cognitive behavioral therapy protocol used in the interdisciplinary group for obesity.

**Weeks**	**CBT strategies**	**Description**
1	Preparatory phase	Psychologist's presentation and therapeutic contract
2	Awareness	String dynamics for group member presentation
3	Goal setting	Physical and psychological consequences of obesity
4	Monitoring food intake	Documentary to analyze the quality and the effects of food advertising
5	Self-monitoring	The importance of self-monitoring normalization of eating habits
6	Problem solving	Group problem solving used to deal with sedentary factors
7	Feedback and reinforcement	Self-responsibility in the change process
8	Self-esteem training	Members discuss how the model based on their expectations
9	Assertiveness training	Members discuss progress toward goals, obstacles, and challenges
10	Boosting the belief that you can do it	Daily self-monitoring of food intake and context, thoughts and emotions
11	Stimulus control	Strategies to address the challenges of lifestyle change
12	Incentives	Each member outlines a personalized plan to work toward eating habits
13	Stress management	Anxiety management techniques (discomfort, self-reassurance, relaxation)
14	Relapse prevention	Psychotherapy and autonomy

In an effort to reduce obesity, studies have explored ideal weight loss and weight loss strategies. A 5% weight loss has been shown to improve health results, and is currently a standard goal in many weight loss interventions (12, 13). However, there are a number of barriers that prevent progress in reversing the obesity pandemic, including the limitations of the treatments. Lifestyle interventions that aim to reduce obesity are generally limited to physical activity and/or nutrition. Interdisciplinary approaches that consider the multifactorial roots of obesity and include psychological treatments are required to increase the effectiveness of interventions.

It is essential to develop more powerful strategies to address this obesity epidemic and help individuals lose weight, as well as assist them in adopting and maintaining a healthy lifestyle in a “toxic” environment, that promotes excessive food consumption, advocating the development of a positive energy balance. In fact, studies have demonstrated the efficacy of interdisciplinary programs in developing a healthy lifestyle, and shown how understanding the psychological factors involved in obesity can both broaden the theoretical framework and contribute to the development of improved obesity treatment interventions (14, 15).

In this context, the present study aimed to analyze and compare the three different types of treatment described above, and divided the sample into three groups: the education and health group (EH), the physical exercise group (PE), and the interdisciplinary therapy plus cognitive behavioral therapy group (IT + CBT).

### 1.1. Study Protocol

This study hypothesized that the three interventions would bring about different beneficial health results; We hypothesized that (1) individuals in the EH group would show greater increases in quality of life in general; (2) those in the PE group would show greater increases in physical activity, flexibility, and conditions physic-related health; and (3) the participants in the IT + CBT group would engage in a higher number of scheduled target activities, change lifestyle behaviors, and present greater weight loss. This study was characterized as a quantitative and qualitative research survey and had a randomized clinical trial format.

## 2. Methods

### 2.1. Design of the Study

**Figure 1:**
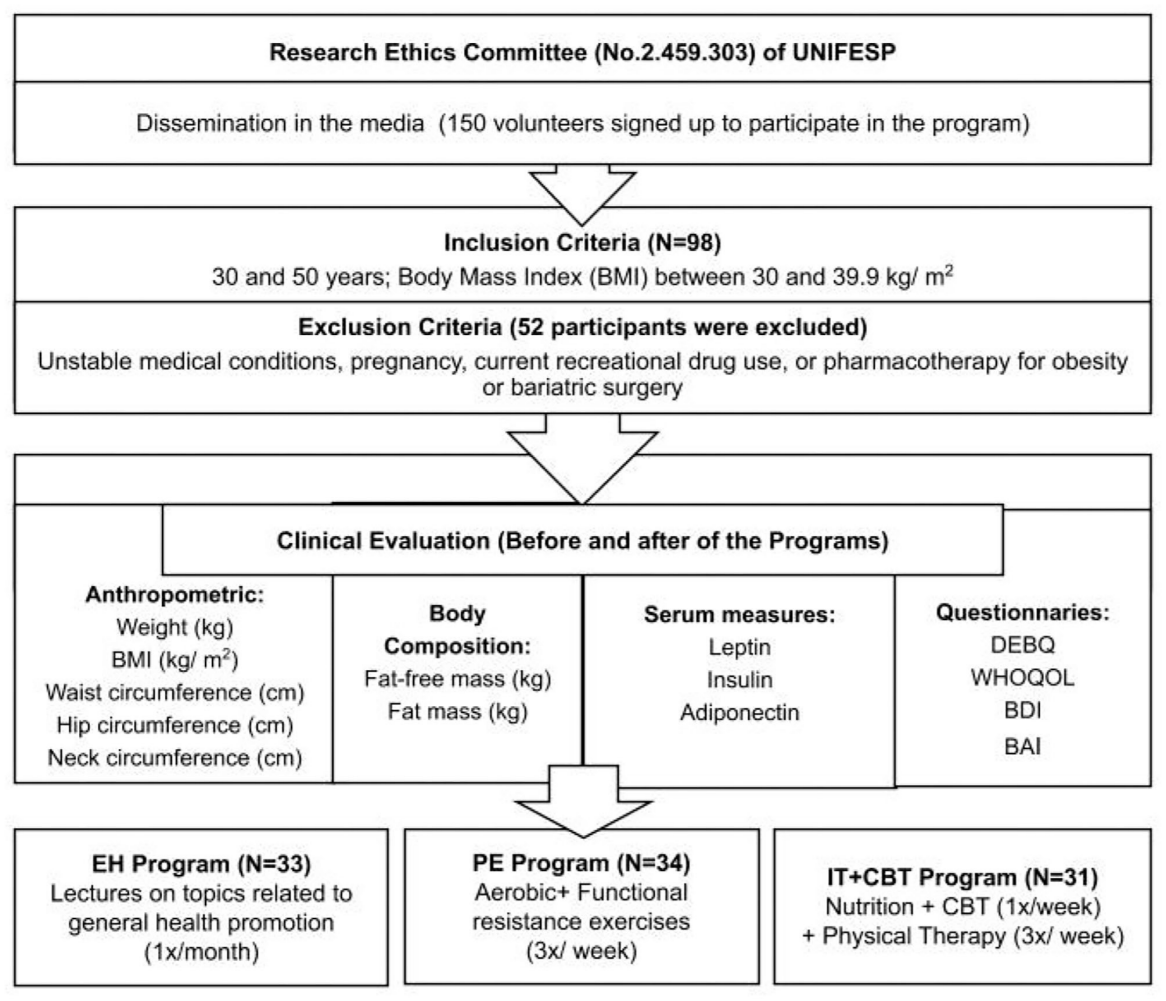
BMI, Body mass index (BMI); DEBQ, Dutch Eating Behavior Questionnaire; WHOQOL-Brief, World Health Organization quality of life instrument; BAI, Beck Anxiety Inventory; BDI, Beck Depression Inventory (BDI); EH, education and health; PE, physical exercise; IT+CBT, interdisciplinary therapy plus cognitive behavioral therapy.

Body mass index (BMI); Dutch Eating Behavior Questionnaire (DEBQ); World Health Organization quality of life instrument (WHOQOL-Brief); Beck Anxiety Inventory (BAI); Beck Depression Inventory (BDI); education and health (EH); physical exercise (PE); interdisciplinary therapy plus cognitive behavioral therapy (IT+CBT) (see Figure 1).

### 2.2. Participants

The volunteers were recruited by the Obesity Study Group, an interdisciplinary team established at the university to address problems of overweight and obesity among patients at the Federal University of São Paulo, Santos/SP, Brazil, through local community advertising in February 2017. The inclusion criteria were as follows: males and females aged between 30 and 50 years, and with a body mass index (BMI) between 30 and 39.9 kg/m^2^. The exclusion criteria were as follows: unstable medical conditions (e.g., cardiovascular disease, cardiopulmonary problems, and psychiatric illness), pregnancy, current recreational drug use or pharmacotherapy for obesity, or having undergone bariatric surgery. All participants were required to sign an informed consent form and agree to participate voluntarily in the research. The study was approved by the Research Ethics Committee (No. 2.459.303) of UNIFESP and was registered with the Clinical Trial database, registration number NCT02573688. The procedures were conducted in compliance with the Declaration of Helsinki principles and the standards established by Brazilian Legislation in Resolution No. 466/2012 of the National Health Council and obeyed the Criteria of Ethics in Research with Human Beings according to Resolution number 466 of the National Health Council.

Of the 150 volunteers who initially agreed to participate in the program, 52 were excluded after applying the study criteria, leaving a total of 98 adults with obesity. They were randomly (through a Microsoft Excel specific function) assigned to one of the three groups: HE, PE, and IT + CBT. Demographic data, including age and sex, history of obesity, and socioeconomic status, were collected (see Table 2). All the volunteers were evaluated before and after the programs, with anthropometric measures, and metabolic parameters through blood sampling being recorded. The volunteers also self-completed a number of questionnaires, with the help of a health professional trained to apply each questionnaire. Results were compared within and between groups. The study was conducted at the Interdisciplinary Laboratory of Metabolic Diseases at the Federal University of São Paulo, Santos, Brazil.

**Table 2:** Comparison of participants' characteristics by group before the programs.

**Characteristics of volunteers**	**EH (33)**	**PE (34)**	**IT+CBT (31)**
Age, Mean (SD)	35.98 ± 6.76	37.97 ± 5.96	36.18 ± 2.75
Female (%)	27 (81.8%)	28 (82.4%)	24 (77.4%)
Male (%)	6 (18.2%)	6 (17.6%)	7 (22.6%)
**Schooling**			
Completed High School (%)	9 (27.3%)	5 (15%)	9 (29.0%)
Bachelors Degree (%)	22 (66.7%)	29 (85%)	22 (71.0%)
Non-specific (%)	2 (6.0%)	0 (0%)	0 (0%)
**Marital status**			
Single (%)	14 (42.4%)	11 (32.4%)	11 (35.5%)
Married (%)	18 (54.5%)	23 (67.6%)	15 (48.4%)
Divorced (%)	1 (3.0%)	0 (0%)	2 (6.4%)
Widower (%)	0 (0%)	0 (0%)	0 (0%)
Stable union (%)	0 (0%)	0 (0%)	3 (9.7%)
**Weight loss strategy**			
Diet (%)	11 (33.3%)	14 (41.2%)	16 (51.6%)
Physical activity (%)	4 (12.1%)	8 (23.5%)	8 (25.8%)
Medicines (%)	13 (39.4%)	11 (32.3%)	11 (35.5%)
Not specific (%)	15 (45.4%)	12 (35.3%)	10 (32.2%)
**Attribution of obesity**			
Hereditary (%)	10 (30.3%)	9 (26.5%)	8 (25.8%)
Medicines (%)	1 (3.0%)	4 (11.8%)	4 (12.9%)
Eating habits (%)	24 (72.7%)	25 (73.5%)	19 (61.3%)
Sedentary lifestyle (%)	22 (66.6%)	26 (76.5%)	17 (54.8%)
Anxiety/depression (%)	19 (57.6%)	19 (55.9%)	18 (58.0%)
Not specific (%)	5 (15.1%)	7 (20.6%)	7 (22.6%)
**Diagnosis**			
Systemic arterial hypertension (%)	10 (30.3%)	6 (17.6%)	4 (12.9%)
Stroke (%)	2 (6.1%)	0 (0%)	0 (0.0%)
Hypercholesterolemia (%)	9 (27.3%)	11 (32.4%)	5 (16.1%)
Diabetes (%)	2 (6.1%)	2 (5.9%)	3 (9.7%)
Myocardial infarction (%)	0 (0.0%)	0 (0%)	0 (0.0%)
Thyroid disorders (%)	11 (33.3%)	6 (17.6%)	3 (9.7%)
Eating disorder (%)	0 (0.0%)	0 (0%)	0 (0.0%)
Psychological disorder (%)	1 (3.0%)	2 (5.9%)	3 (9.7%)
**Family diagnoses**			
Obesity (%)	20 (60.6%)	22 (64.7%)	19 (58.1%)
Systemic arterial hypertension (%)	24 (72.7%)	23 (67.6%)	26 (77.4%)
Stroke (%)	8 (24.2%)	4 (11.8%)	9 (77.4%)
Hypercholesterolemia (%)	16 (48.5%)	20 (58.8%)	20 (61.3%)
Diabetes (%)	12 (36.4%)	19 (55.9%)	17 (51.6%)
Myocardial infarction (%)	7 (21.2%)	18 (52.9%)	10 (29.0%)
Thyroid disorders (%)	8 (24.2%)	9 (26.5%)	12 (35.5%)
Smokers (%)	2 (6.0%)	6 (17.6%)	1 (3.2%)
**When most hungry**			
Night (%)	11 (33.3%)	9 (26.5%)	18 (58.1%)
When anxious (%)	2 (6.1%)	0 (0.0%)	0 (0.0%)
Not specific (%)	5 (15.2%)	4 (11.8%)	5 (16.1%)
Lunch (%)	5 (15.2%)	2 (5.9%)	3 (9.7%)
Afternoon (%)	2 (6.1%)	13 (38.2%)	3 (9.7%)
Morning (%)	6 (18.2%)	6 (17.6%)	2 (6.5%)
Anytime (%)	2 (6.1%)	0 (0.0%)	0 (0.0%)
**Preferred type of food**			
Candy (%)	11 (33.3%)	20 (58.8%)	14 (41.9%)
Not specific (%)	7 (21.2%)	6 (17.6%)	9 (25.8%)
Junk food (%)	9 (27.3%)	4 (11.8%)	0 (0.0%)
Bread (%)	1 (3.0%)	2 (5.9%)	2 (6.5%)
Pasta (%)	3 (9.1%)	1 (2.9%)	0 (0.0%)
Rice (%)	0 (0.0%)	1 (2.9%)	0 (0.0%)
Mother's food (%)	0 (0.0%)	0 (0.0%)	1 (3.2%)
Meat (%)	0 (0.0%)	0 (0.0%)	1 (3.2%)
Ice Cream (%)	0 (0.0%)	0 (0.0%)	1 (3.2%)

### 2.3. Materials

#### 2.3.1. Questionnaires

**Depression and Anxiety Symptoms:** Depression symptoms were assessed using the Beck Depression Inventory (BDI), and anxiety symptoms were assessed using the Beck Anxiety Inventory (BAI). Both inventories have been translated into Portuguese and validated for use with the Brazilian population (16).

**Eating Behavior Assessment:** The Dutch Eating Behavior Questionnaire (DEBQ) (17) evaluates emotional, external, and restrained eating behaviors (18). This instrument has been validated for use in Brazil (19).

**Quality of Life Assessment:** The quality of life of each participant was evaluated using the World Health Organization quality of life instrument (WHOQOL-Brief), translated into Portuguese and validated for the Brazilian population (20). The WHOQOL-Brief consists of 26 questions (two general questions that assess quality of life and 24 questions that represent specific items in the physical, psychological, social, and environmental domains).

#### 2.3.2. Anthropometric Measurements

The volunteers wore light clothing without shoes when all anthropometric measures were taken. A Toledo balance with a maximum capacity of 200 kg and a calibration of 0.01 kg was used to determine body mass, and a wall-mounted stadiometer was used to measure height to the nearest 0.5 cm. Having obtained the data, the ratio of the weight to the height squared (kg/m^2^) was used to calculate the BMI. The measures of the waist (WC), thigh (TC), and hip circumferences (HC) were taken using a flexible inelastic tape without compressing the tissue. WC was measured at the midpoint between the iliac crest and last rib at the natural end of an exhalation (21). HC was determined at the greatest extension of the buttocks.

#### 2.3.3. Metabolic Parameters

At about 8:00 a.m., after overnight fasting (12 h), blood samples were collected at the outpatient clinic. For the tests, about 30 ml of venous blood was removed, and the blood was centrifuged for 10 min at 3, 500 rpm and frozen at 80°*C* for future analyses. Serum leptin concentrations were measured by a commercially available radioimmunoassay kit (Human Leptin DuoSet ELISA Kit; R&D Systems^®^, Minneapolis, MN, USA). Serum adiponectin concentration was determined by enzyme linked immunosorbent assay (Human Total Adiponectin/Acrp30 DuoSet ELISA; R&D Systems^®^). Insulin was analyzed by the ELISA method using a commercial kit (ELISA commercial kit, Human Insulin ELISA, 96-well strip plate; Millipore^®^, Billerica, MA), following all the manufacturer's recommendations.

### 2.4. Clinical Intervention

**Education and Health Program (EH):** Health professionals gave one lecture per month to advise volunteers on lifestyle, on the following key topics: (1) motivation to change behaviors; (2) psychological aspects related to weight loss; (3) healthy eating; (4) physical therapy care in the process of losing weight and obesity; (5) obesity-associated diseases; (6) physical exercise to improve health and lose weight; and (7) bariatric surgery. During the investigation, the researchers kept in contact with the volunteers using WhatsApp, and the members of the EH group were connected to each other using WhatsApp^®^ group chat to share information about therapy and feedback on lectures/meetings.

**Physical Exercise Program (PE):** The participants underwent a training protocol, which consisted of performing exercises that synergistically integrated and balanced different components of physical fitness (22). This was a group training protocol but the control and progression of the load was individual. The environment for the intervention was a climate-controlled (~20 to 22°*C*) space equipped with ergometers (bicycle and treadmills) and accessories for resistance training. The PE program volunteers underwent sessions three times a week (Monday, Wednesday, and Thursday) including aerobic and functional resistance exercises. The training session lasted 60 min and comprised the following elements: warm-up (5 min), followed by aerobic exercise (25 min) plus functional resistance exercises (25 min), with the participants allowed to change the order of the sequence, and finishing with relaxation (5 min). An ergometer (treadmill or bicycle) was used to apply the aerobic exercises at an intensity equivalent to the rating of perceived exertion (RPE) of 13–14 on the Borg scale (23). A circuit scheme with 3 passes in 8 stations was used to perform the resistance training. Each pass consisted of a 40 s execution with a 20 s passive interval. A total of four different circuits was developed, which followed a monthly rotation. Free weights (FW—bars, plates, dumbbells, poles), elastic bands (EB), and bodyweight (BW) were used for overload of the exercises. Only the fourth circuit was based on manual resistance training (MRT), as suggested by La Scala Teixeira et al. (24).

The IT + CBT group underwent a physical exercise training program identical to the PE group. On each day of physical training, however, interventions on nutrition, psychology, and physical therapy were added once a week as part of an interdisciplinary approach. In order to strengthen the interdisciplinary character of the proposed treatment, an intervention between two or more fields was developed once a month.

Psychological intervention: Once a week 60-min sessions of psychology interventions were performed. CBT was the underlying theoretical approach used to guide the psychological intervention. Cognition has an important role in the expression of feelings and behavior in this model. A more realistic and adaptive perspective is achieved as the individual learns how to detect and react to dysfunctional thoughts, promoting a better emotional state and more adaptive behavior to one's environment (25).

The psychological intervention consisted of 14 one-hour sessions with a psychologist, adapted of the protocol of the White and Freeman (26), to Cognitive-Behavioral group therapy for specific problems and population.

The first part and the second parts followed the same protocol (each one with 14 themes) in addition to two interdisciplinary interventions, one being psychological and nutritional relating to emotional eating behaviors and the other psychological and physical and relating to relaxation strategy. The two strategies comprised the 30 weeks interventions.

**Nutrition intervention:** A dietary guide for the population (27) and the principles of behavioral nutrition provided the base for the interventions, as well as specific information about food, nutrition, and dietetics. The contents treated were transmitted weekly 60-min sessions, mainly using expository and talking methods. Furthermore, every 2 months the practical cooking knowledge of the participants was enhanced by practical classes in experimental cooking, giving the volunteers the opportunity to have practical gastronomical and nutritional experience. At the outset of the therapy, an individualized dietary plan was designed according to the energetic and nutritional needs of each participant, serving as a helpful tool to make daily food choices for those who had difficulties in respect of quantities and portion sizes of food.

**Physical therapy intervention:** The group experienced a physical exercise training program equal to the PE group. Physical therapy interventions aimed to improve the volunteers' functional capacity. Each session lasted 60 min including postural exercises, lumbopelvic stabilization (core training), dynamic balance, and flexibility exercises (24).

### 2.5. Data Analysis Procedure

The quantitative data analysis was performed using the software R-version 3.6.0. (28). The normality of the variables was verified using the Shapiro–Wilk test. Parametric data were presented as mean ± standard deviation (SD). The Wilcoxon test for paired samples was used to compare responses at the pre- and post-intervention moments. To compare the responses between the groups, the Kruskal–Wallis test was applied for each moment (pre- and post-intervention), also considering the difference in responses between the pre- and post-intervention moments.

To verify whether the composition of the three groups was homogeneous following the intervention, we chose to analyze whether the differences between the groups at the beginning of the study were statistically significant using the Kruskal–Wallis test. The test showed that there is no evidence to reject the hypothesis that the distribution of variables is the same for the Education and Health, Physical Exercise, Interdisciplinary plus Cognitive and Behavior Therapy programs, at a significance level of 5% (the *p*-value of the test is greater than 5% for all variables analyzed). All statistical tests were two-tailed, with a significance level 0.05 for normality tests and simple comparisons. For *Post-hoc* analysis, the pairwise Mann–Whitney test with the Bonferroni procedure was used, and the adjusted *p*-value was set at 0.0167.

## 3. Results

After 30 weeks, 43 participants remained until the end of treatment: EH (*N* = 12), PE (*N* = 13), IT+CBT (*N* = 18); among them, the following were with minimum number of frequency in the interventions (70%): EH (77.9±11.7), PE (78.0±9.3), and IT + CBT (75.7±10.7). The dropout rate over the period of 30 weeks was lower for the IT + CBT group (41.9%), (~20%) than for the other groups, confirming the effectiveness of the intervention, compared with the others two groups, EH (63.6%) and PE (61.8%).

The sample was predominantly female in all the programs. This was to be expected as it has been shown that women are more likely to seek obesity treatment than men because they are not satisfied with their physical appearance and also because they are more concerned about the associated health problems (29, 30).

###  Behavioral Factors

The IT + CBT program promoted greater behavioral changes than the other two programs in factors relevant to weight control, such as activity level and dietary intake, resulting in more weight loss. Changes in almost all the variables investigated were observed, including reductions in weight, body mass index, absolute fat mass, waist circumference, hip circumference, and neck circumference. The program was shown to be effective in increasing quality of life in all the domains (physical, psychological, social, environmental), and reducing symptoms of depression and emotional, external and restricted eating behaviors (Table 3).

**Table 3:** Comparison of pre- and post-intervention variables for each group by Wilcoxon test for paired samples in obese adults.

**Variables**	**EH**			**PE**			**IT+CBT**			
	**Before**	**After**	***p***	**Before**	**After**	***p***	**Before**		**After**	***p***
Body weight (kg)	94.88 ± 10.61	92.14 ± 12.53	0.20	92.63 ± 13.19	91.21 ± 12.57	**0.03**	92.56 ± 12.16		89.49 ± 12.99	**0.00**
BMI (kg/m^2^)	35.69 ± 2.57	34.65 ± 3.55	0.18	34.42 ± 2.70	33.91 ± 2.68	**0.03**	35.57 ± 2.85		34.12 ± 3.18	**0.00**
Waist circumference (cm)	102.83 ± 8.71	100.03 ± 11.27	0.11	104.02 ± 10.36	101.12 ± 8.06	**0.04**	103.54 ± 7.40		98.74 ± 8.86	**0.00**
Hip circumference (cm)	121.46 ± 9.28	118.20 ± 8.90	0.05	120.22 ± 9.57	117.08 ± 9.91	**0.00**	119.53 ± 10.06		115.97 ± 9.25	**0.00**
Neck circumference (cm)	36.48 ± 2.20	36.13 ± 2.44	0.72	36.87 ± 2.39	35.92 ± 2.12	**0.04**	36.41 ± 2.18		35.15 ± 1.97	**0.00**
Fat-free mass (kg)	56.75 ± 6.25	55.38 ± 5.69	0.09	55.37 ± 6.16	54.14 ± 5.83	0.13	54.11 ± 6.13		53.38 ± 5.96	0.07
Fat mass (kg)	38.13 ± 5.93	36.75 ± 8.13	0.38	37.27 ± 8.84	37.07 ± 7.80	0.74	38.45 ± 6.54		36.11 ± 7.69	**0.02**
Leptin (ng/ml)	56.97 ± 31.62	50.56 ± 27.39	0.46	52.66 ± 24.86	50.77 ± 25.64	0.20	67.47 ± 28.80		59.65 ± 35.06	0.18
Adiponectin (g/l)	2.35 ± 0.75	2.50 ± 0.94	0.41	2.09 ± 1.08	2.04 ± 1.12	1.00	2.50 ± 0.91		2.68 ± 2.29	0.67
Insulin (ng/ml)	9.35 ± 4.83	7.24 ± 5.36	0.49	12.71 ± 12.12	11.70 ± 8.41	0.59	13.47 ± 14.76		11.88 ± 8.70	0.56
WHOQOL total	10.67 ± 1.97	12.67 ± 1.56	**0.04**	11.38 ± 2.22	12.15 ± 2.23	0.07	9.67 ± 2.20		11.00 ± 1.63	0.07
WHOQOL Physic	13.00 ± 2.52	12.14 ± 2.41	0.09	11.74 ± 1.23	12.79 ± 1.20	**0.02**	11.62 ± 1.36		13.32 ± 1.14	**0.00**
WHOQOL psychic	13.00 ± 1.29	12.67 ± 1.06	0.47	12.51 ± 1.31	12.92 ± 2.01	0.47	12.26 ± 1.77		13.50 ± 1.55	**0.02**
WHOQOL Social	14.56 ± 3.29	14.33 ± 3.78	0.67	15.08 ± 3.63	15.38 ± 3.38	0.80	13.70 ± 2.66		15.50 ± 1.88	**0.00**
WHOQOL Environment	13.63 ± 2.22	13.96 ± 2.14	0.40	14.12 ± 2.07	14.23 ± 2.06	0.76	12.81 ± 1.89		14.00 ± 2.03	**0.01**
DEBQ total	18.42 ± 4.32	18.92 ± 3.90	0.84	20.46 ± 6.60	18.92 ± 7.34	0.10	20.39 ± 5.67		12.00 ± 5.59	**0.00**
DEBQ Restrict	4.92 ± 2.50	4.42 ± 2.61	0.62	5.85 ± 3.11	4.62 ± 2.96	0.11	5.06 ± 2.31		3.19 ± 1.68	**0.01**
DEBQ Emotional	7.58 ± 3.90	8.75 ± 3.36	**0.01**	8.08 ± 4.31	7.77 ± 4.88	0.61	8.72 ± 3.41		4.50 ± 3.71	**0.00**
DEBQ External	6.50 ± 2.07	6.25 ± 2.22	0.72	7.31 ± 2.53	7.08 ± 3.17	0.51	7.11 ± 2.35		4.56 ± 2.92	**0.00**
BDI	16.00 ± 6.05	11.88 ± 5.87	0.36	15.17 ± 9.26	10.27 ± 9.48	1.00	16.94 ± 8.04		6.81 ± 4.93	**0.01**
BAI	11.50 ± 7.71	14.88 ± 12.37	1.00	13.83 ± 9.82	11.00 ± 9.71	1.00	10.72 ± 4.65		6.31 ± 6.48	0.09

*BMI, Body mass index; DEBQ, Dutch Eating Behavior Questionnaire; WHOQOL-Brief, World Health Organization quality of life instrument; BAI, Beck Anxiety Inventory; BDI, Beck Depression Inventory; EH, education and health; PE, physical exercise; IT + CBT, interdisciplinary therapy plus cognitive behavioral therapy. Values shown as mean and standard deviation, p < 0.05. The meaning of the bold values shown as p < 0.05*.

Each of the programs presented improvements, but in comparison with the IT+CBT program, the latter, as expected, presents more benefits than the others.

**Figure 2:**
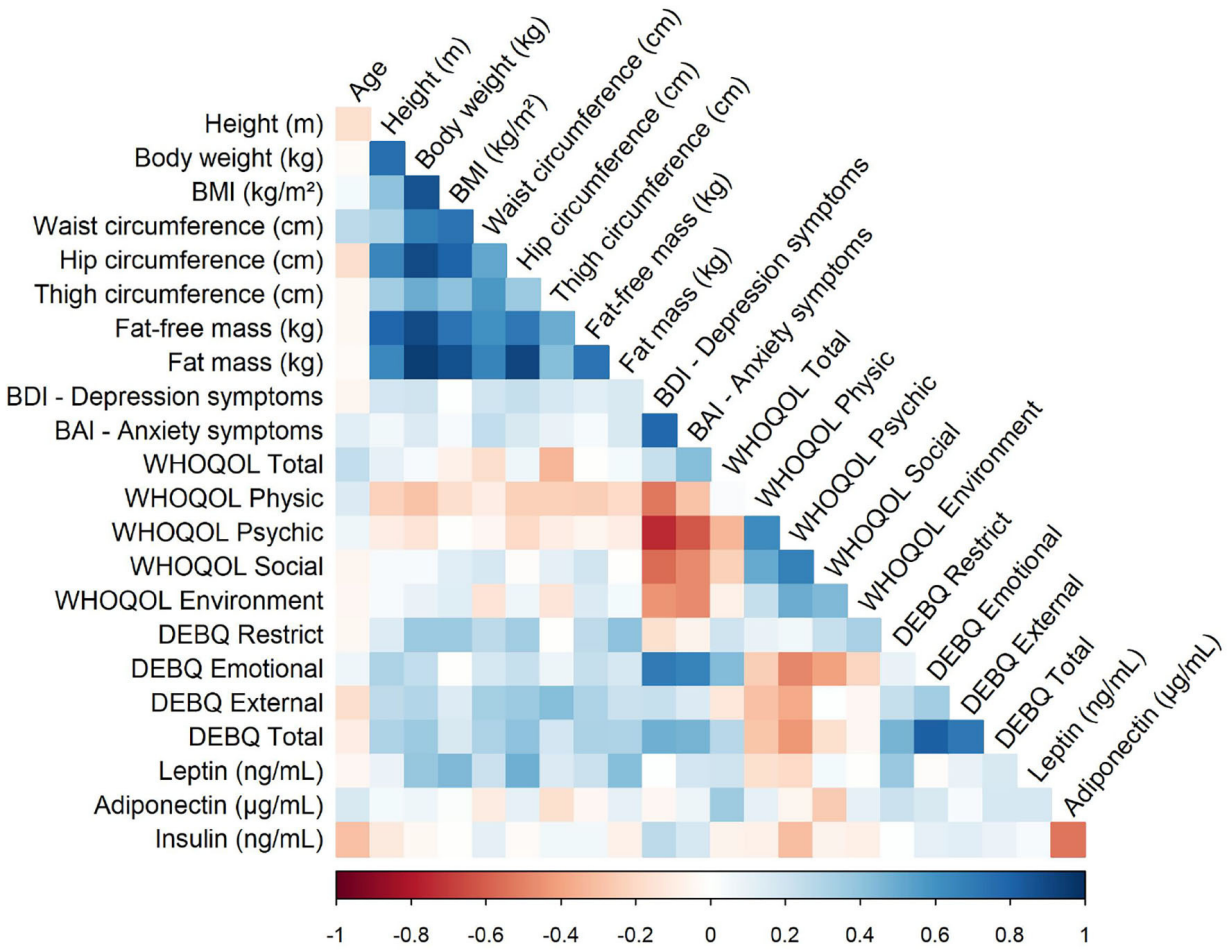
Correlation of values between variables. In figure, the blue indicates positive and red negative correlations. The x shows the correlations that were not significant. Values shown as mean and standard deviation, *p* < 0.05. BMI, Body mass index; DEBQ, Dutch Eating Behavior Questionnaire; WHOQOL-Brief, World Health Organization quality of life instrument; BAI, Beck Anxiety Inventory; BDI, Beck Depression Inventory; EH, education and health; PE, physical exercise; IT + CBT, interdisciplinary therapy plus cognitive behavioral therapy.

As expected, a positive correlation was found in all groups between changes in depression and anxiety symptoms and emotional eating behavior. Furthermore, the perception of physical quality of life was correlated negatively with depression and anxiety symptoms (Figure 2).

In all groups, we found a positive correlation between body weight and hip circumference; body weight and fat mass (kg); emotional eating and anxiety; and emotional eating and depression symptoms. Moreover, we found negative correlations between physical quality of life and depression symptoms; and physical quality of life and anxiety symptoms. Corroborating this correlation, obesity has a substantial impact on an individual's HRQOL (9).

The study revealed that environmental quality of life and BMI were negatively correlated in the EH program. de Oliveira Lima et al. (30) reported that the association of individual and environmental factors determines QOL, emphasizing the importance of lifestyle changes and the effect of the built environment on access to places that may or may not encourage healthy eating and the practice of physical activity.

In the PE program, we found a positive correlation between insulin and depression symptoms. Milaneschi et al. (31) in a recent review described the depression–obesity link, including alterations in homeostatic adjustments. Insulin dysregulation may well represent a mediating mechanism in the obesity–depression relationship, and is highly influenced by environmental factors. In the same program, we found negative correlations between physical quality of life and fat mass (kg). Exercise is an integral part not only of weight loss, but also of overall health (32). Using exercise to reduce obesity has benefits beyond the reduction of fat mass, such as decreasing the comorbidities associated with the obesity.

**Figure 3:**
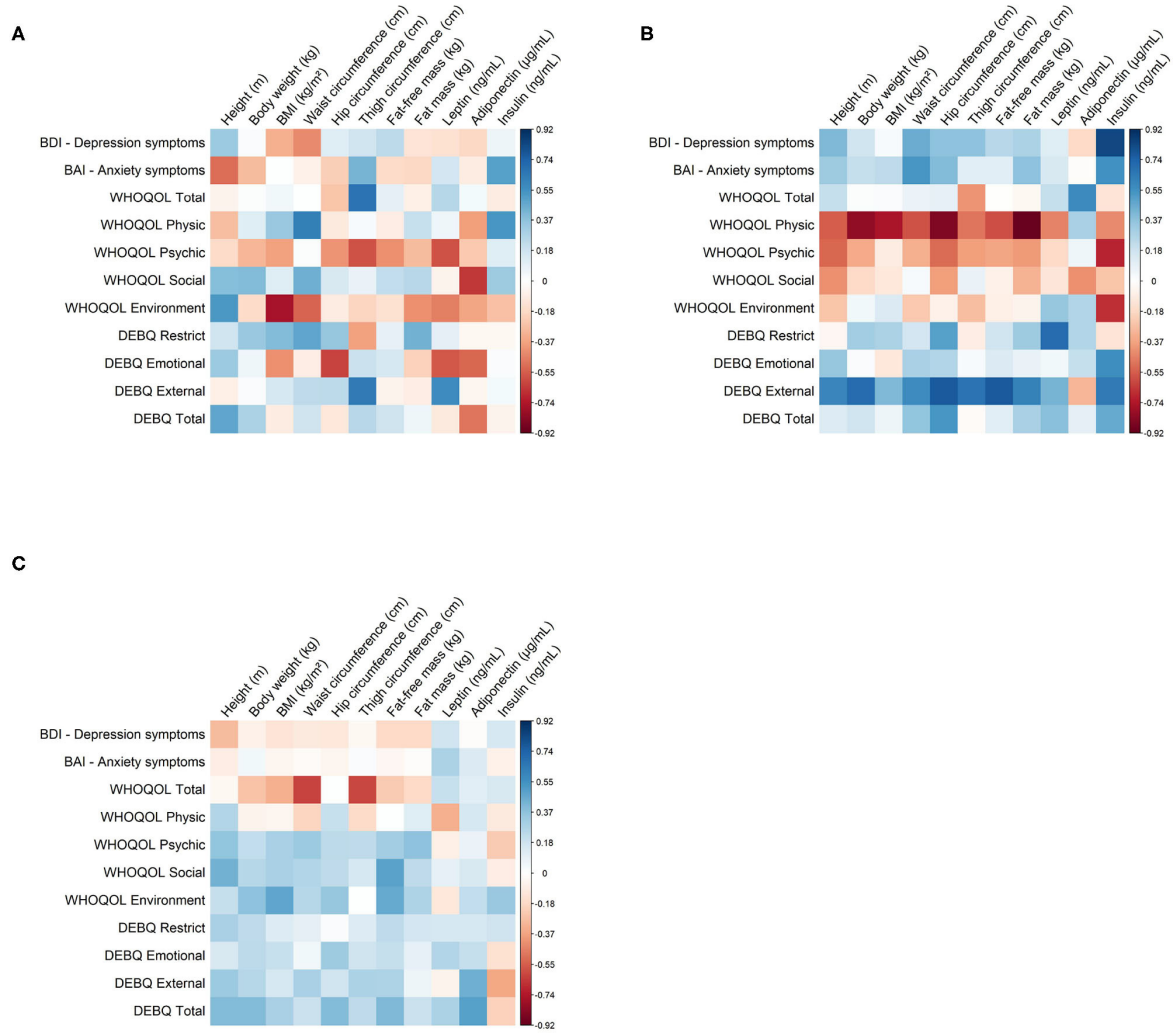
Correlation of values between variables of the three programs (EH, PE and IT+CBT). In the figure, blue indicates to positive and red negative correlations. The x shows the correlations that were not significant. Values shown as mean and standard deviation, *p* < 0.05. BMI, Body mass index; DEBQ, Dutch Eating Behavior Questionnaire; WHOQOL-Brief, World Health Organization quality of life instrument; BAI, Beck Anxiety Inventory; BDI, Beck Depression Inventory; EH, education and health; PE, physical exercise; IT + CBT, interdisciplinary therapy plus cognitive behavioral therapy. **(A)** EH program. **(B)** PE program. **(C)** IT + CBT program.

As expected, in the IT + CBT program, total quality of life and waist circumference, and total quality of life and thigh circumference were inversely correlated. In a recent study, BMI and psychological symptoms were found between the main determinants of HRQoL (33) (see Figure 3).

## 4. Discussion

Our main finding was that the various weight management interventions applied in this study influenced the analyzed variables differently, confirming the initial hypothesis. The IT + CBT program improved the quality of life, eating behaviors, anthropometric profiles, and decreased depression symptoms in adults with obesity. These findings confirm the results of previous studies and are line with what literature recommends as the “gold standard” for obesity treatment (8).

In this context, the World Health Organization (The WHOQOL Group, 1995) defined quality of life as “an individual's perception of their position in life in the context of the culture and value systems in which they live and in relation to their goals, expectations, standards and concerns,” and this involves physical, psychological, independence level, social relationships, and environmental domains. As observed in the results, the level of the quality of life in the IT + CBT program increased in all the domains of the WHOQOL (physical, psychological, social, and environmental). In effect, behavior changes were motivated by the sharing of information between the researchers and participants, mainly demonstrated by altered attitudes toward eating patterns and physical activity.

Dysfunctional beliefs act as cognitive “traps” in the thinking processes of obese individuals, and can be attributed to weight, diet, and personal worth (34).

It is believed that IT + CBT favored the adherence to the treatment and the development of more flexible cognitive patterns that may have helped the effectiveness of the behavior changes (35). Self-monitoring of food intake and physical activity were the core parts shared in respect of the psychological interventions of the IT + CBT program (36).

It is likely that the integrated actions in health such as those observed through cognitive restructuring were helpful for patients by allowing them to question problematic thoughts that interfere with the adherence to lifestyle changes. These results show the importance of cognitive control in behavior changes and how interdisciplinary action is more effective than using a single approach.

The interdisciplinary therapy used with the CBT was designed to reflect the challenges of daily life, in respect of prevention and promotion of health, and concentrated not only on losing weight, but also on the cognitive processes encountered during the treatment of obesity. The treatments that patients with obesity usually undergo are often mainly tailored to deal with biological and behavioral factors that impede weight loss and its maintenance, while ignoring the underlying cognitive processes that may be in effect (37). One of the reasons for the diminished effectiveness of biological and behavioral treatments that focus on long-term weight loss may be a failure to properly address the problems relation to the ability of the patient to adhere to lifestyle modification over time (38).

Since long-term interdisciplinary therapy has higher costs for the obese patient and health services, this study also considered the effectiveness of more inexpensive interventions that are consequently more accessible to a larger population.

The results from the PE program confirm those of a previous study conducted in Brazil that reported that almost 50% of participants with obesity dropped out of isolated physical training programs (24). This shows the importance of combining PE approaches with nutritional and psychologically interventions in order to achieve better results. It is also important to note that our study found that the benefits of the physical exercise are mainly only in respect of the anthropometric measures and the perception of quality of life in the physical domain (see Table 4).

**Table 4:** Comparison of pre- and post-intervention variables for each group using the Kruskal–Wallis test.

**Variable**	**EH (11) △**	**PE (12) △**	**IT+CBT (17) △**	***p*-valor**
Body weight (kg)	−2.74	−1.43	−3.06	0.43
BMI (kg/m^2^)	−1.04	−0.51	−1.45	0.38
Waist circumference (cm)	−2.79	−2.89	−4.8	0.36
Hip circumference (cm)	−3.26	−3.14	−3.56	0.98
Thigh circumference (cm)	−0.35	−0.95	−1.26	0.15
Fat-free mass (kg)	−1.37	−1.23	−0.73	0.72
Fat mass (kg)	−1.37	−0.20	−2.34	0.32
Leptin (ng/ml)	−6.41	−1.90	−7.82	0.94
Adiponectin (μg/l)	0.16	−0.05	0.18	0.56
Insulin (ng/ml)	−2.11	−1.01	−1.60	0.98
WHOQOL total	2, 00	0.77	1.33	0.69
WHOQOL Physic	−0.86	1.05	1.70	0.00^*ab*^
WHOQOL Psychic	−0.33	0.41	1.24	0.04
WHOQOL Social	−0.22	0.31	1.80	0.02
WHOQOL Environment	0.33	0.12	1.19	0.01^*c*^
DEBQ Total	0.50	−1.54	−8.39	0.00^*bc*^
DEBQ Restrict	−0.50	−1.23	−1.87	0.53
DEBQ Emotional	−1.17	−0.31	−4.22	0.00^*bc*^
DEBQ External	−0.25	−0.23	−2.55	0.00^*bc*^
BDI	−4.12	−4.90	−10.13	0.02
BAI	3.38	−2.83	−4.41	0.12

*Post-hoc pairwise Mann–Whitney. a: comparison between EH and PE Program, p < 0.0167; b: comparison between EH and IT + CBT Program, p < 0.0167; c: comparison between IT + CBT and PE program, p < 0.0167*.

On other hand, the results of the EH program showed that there was a positive effect on the total of quality of life variables in the samples that completed the intervention, but the dropout rate over the intervention period was high. The patients were motivated by the information transmitted through the lectures to make changes to their lifestyle behaviors. In this context, the quality of life of the HE participants significantly improved, but emotional eating behavior increased.

Emotional eating refers to a tendency to eat in response to negative emotions (e.g., depression, anxiety, and stress) with the chosen foods being primarily energy-dense and palatable ones (39). It can be caused by various mechanisms, such as using eating to cope with negative emotions, or confusing internal states of hunger and satiety with physiological changes associated with emotions (40). Unlike the IT+CBT group, the volunteers in the EH group did not receive interventions explaining how to learn to cope with negative emotions, and how not to confuse internal states of hunger and satiety, and this may explain the different results in the two programs. Therefore, we believe that although health education is important, the cognitive aspects have to be considered in obesity treatment. It is interesting to note that higher physical activity has also been linked with lower emotional eating (40, 41).

According to Minayo (42), qualitative research is understood as research that is concerned with the subjective and relational level of social reality and that deals with history, the universe of meanings, motives, beliefs, values, and the attitudes of social actors.

Therefore, we describe three testimonials from volunteers after the interventions: “After the lectures, I started to look at food labels, and to be more aware of the choices of foods that are good for health. I also became more conscious of not doing enough exercise, and even ate more regularly. Sometimes I used the breathing techniques that I learned in the lecture” (S. volunteer, EH program). “I noticed changes in weight and measurements, I felt more free every day, and I find it easier to perform tasks. I feel more willing to do activities, I feel less pain in my body and I started to take more care of myself” (M volunteer, PE program). “After the interventions I started to exercise, my quality of sleep improved, I have greater flexibility, and a better mood I feel less anxious and agitated, with better self-esteem and started to think before eating and assessing what I'm eating, I feel less tired and more balanced, even my gut is working better” (E. volunteer, IT + CBT program).

In terms of limitations, although there is good evidence for the effectiveness of multi-disciplinary approaches, long-term results are often limited. This approach is commonly restricted by high costs and, therefore, availability. This particular study was limited by the large sample dropout rate over the period of the intervention. However, overweight and obese people in long-term lifestyle modification programs do tend to have a high dropout rate, being higher than in studies with non-obese individuals, as has been shown in previous studies (43). Consequently, researchers and professionals dealing with this population will have to give greater attention to this challenge. Another important limitation this study does not included other metabolic markers and cardio-vascular risk management. The physical exercise has benefits on cardiorespiratory health in overweight people (44).

The IT + CBT program, when compared with the physical exercise and education and health programs, presented better results on the perception of volunteers life quality, anthropometric measurements, and decreased depression symptoms, although no changes were observed at the hormonal levels and anxiety symptoms. Our findings highlight the need to expanding the investigation of anxiety and also the inflammation in this population, both in respect of the form of assessment and the treatment of obese adults. Such findings illustrate the complexity of the psychological variables involved in the long-term treatment of obesity.

In conclusion, the results of the study suggest that health-related quality of life and psychological issues may be key elements in the treatment of adults with obesity, and addressing these factors may contribute to advances in clinical actions.

## Data Availability Statement

The raw data supporting the conclusions of this article will be made available by the authors, without undue reservation.

## Ethics Statement

The studies involving human participants were reviewed and approved by the Research Ethics Committee (No. 2.459.303) of UNIFESP and the Clinical Trial Registration Number NCT02573688 database. The patients/participants provided their written informed consent to participate in this study.

## Author Contributions

DC and AM conceived and designed the study. CL, MC, SG, BP, and GS performed the experiments. AM wrote the paper. RG performed the statistic analysis. LO performed laboratory analysis. RP, RG, and AD participated in revising the paper.

## Conflict of Interest

The authors declare that the research was conducted in the absence of any commercial or financial relationships that could be construed as a potential conflict of interest.

## Abbreviations

BAI: Beck Anxiety Inventory
BDI: Beck Depression Inventory
BMI: Body Mass Index
BW: Body Weight
CBT: Cognitive-Behavioral Therapy
DEBQ: Dutch Eating Behavior Questionnaire
EH: Education and Health
EB: Elastic Bands
ELISA: Enzyme-linked Immunosorbent Assay
UNIFESP: Federal University of São Paulo
FW: Free weights
HC: Hip Circumferences
HRQOL: Health-related quality of life
IT + CBT: Interdisciplinary Therapy with Cognitive Behavioral Therapy
PE: Physical Exercise
MRT: Manual Resistance Training
TC: Thigh Circumferences
WC: Waist circumferences
WHO: World Health Organization
WHOQOL-Bref: World Health Organization Quality of Life Instrument.
